# Glances at the Hospitals

**Published:** 1904-12-17

**Authors:** 


### GLANCES AT THE HOSPITALS.

### ROYAL WEST HANTS HOSPITAL.

The gross income for the year was £1,658, and the gross expenditure was £1,644, so that the committee are to be congratulated on the results of the year's working. The management of the hospital shows energetic action on someone's part. There is a considerable building debt to pay off; but a bazaar was organised and the pleasing result of this was that a sum of £625 was received to reduce the debt. A conversazione was inaugurated by Mrs. Hubert Peck, and this produced £95, besides widening the circle of supporters, and an annuity of £48 has been received to partly endow a bed in the hospital. One member of the committee collected a sum of £44 towards the cost of a Roentgen rays apparatus. All these things show what can be done when people are in earnest over their work. Further improvements are to be carried out. The heating arrangements are to be overhauled, and an isolation ward and an operating theatre are required. We cannot doubt that the funds for these will be forthcoming. The total number of in-patients during the year was 330, being an increase of 18 on the previous year. The average number of beds occupied was 18, and the average of each patient's stay in the hospital was 22 days. The average cost per bed occupied was £86 10s., but if the expenses of the out-patients' department be deducted the cost would fall to £77 15s. The average cost per patient was £4 9s. 6d., and the average cost per day was 4s. The above particulars prove that the secretarial work is well done, and we may say the same for the medical tables. The columns in these tables show the numbers treated, cured, relieved, and died. Thus they serve a double purpose in enabling those interested in hospital matters to see what is being done in the Boscombe Hospital, and of permitting comparison with other institutions. Of 10 cases of pneumonia, 8 were cured, 1 relieved and 1 died. Of 8 cases of rheumatic fever, 7 were relieved, and 1 cured. The operation for strangulated hernia was performed 6 times, all being successful. The operation for appendicitis was performed 6 times, in all cases successfully. Altogether 241 surgical operations were performed, and anæsthetics were administered 484 times. Narcotile was the agent employed in 178 instances, ether in 123, nitrous oxide in 78, A.C.E. mixture in 47, chloroform in 38, ethyl chloride in 7, chloroform and ether in 4, somnoform in 3, narcotile ether and chloroform in 2, and the A.C.E. mixture and chloroform in 1. There was no death from any of these.

